# Phase Space Reconstruction Based CVD Classifier Using Localized Features

**DOI:** 10.1038/s41598-019-51061-8

**Published:** 2019-10-10

**Authors:** Naresh Vemishetty, Ramya Lakshmi Gunukula, Amit Acharyya, Paolo Emilio Puddu, Saptarshi Das, Koushik Maharatna

**Affiliations:** 1 Department of Electrical Engineering, IIT Hyderabad, Hyderabad, 502285 India; 2 Department of Cardiovascular Sciences, Sapienza University of Rome, Viale del Policlinico 155, I-00161 Rome, Italy; 3 Department of Mathematics, University of Exeter, Cornwall, TR10 9FE UK; 4 School of Electronics and Computer Science, University of Southampton, Southampton, SO17 1BJ UK

**Keywords:** Biomedical engineering, Electrical and electronic engineering

## Abstract

This paper proposes a generalized Phase Space Reconstruction (PSR) based Cardiovascular Diseases (CVD) classification methodology by exploiting the localized features of the ECG. The proposed methodology first extracts the ECG localized features including PR interval, QRS complex, and QT interval from the continuous ECG waveform using features extraction logic, then the PSR technique is applied to get the phase portraits of all the localized features. Based on the cleanliness and contour of the phase portraits CVD classification will be done. This is first of its kind approach where the localized features of ECG are being taken into considerations unlike the state-of-art approaches, where the entire ECG beats have been considered. The proposed methodology is generic and can be extended to most of the CVD cases. It is verified on the PTBDB and IAFDB databases by taking the CVD including Atrial Fibrillation, Myocardial Infarction, Bundle Branch Block, Cardiomyopathy, Dysrhythmia, and Hypertrophy. The methodology has been tested on 65 patients’ data for the classification of abnormalities in PR interval, QRS complex, and QT interval. Based on the obtained statistical results, to detect the abnormality in PR interval, QRS complex and QT interval the Coefficient Variation (CV) should be greater than or equal to 0.1012, 0.083, 0.082 respectively with individual accuracy levels of 95.3%, 96.9%, and 98.5% respectively. To justify the clinical significance of the proposed methodology, the Confidence Interval (CI), the p-value using ANOVA have been computed. The p-value obtained is less than 0.05, and greater F-statistic values reveal the robust classification of CVD using localized features.

## Introduction

Cardiovascular Diseases (CVD) is specified as one of the serious diseases and became the prime cause of human deaths as per the survey of World Health Organization (WHO)^1^, leading to the immense research in detecting the ECG abnormalities. Due to the insufficient access to the primary health care centers and delayed diagnosis by the population in the developing countries results in the high mortality rate. Considering the current lifestyle, there is great necessity to develop a robust algorithm to find any desynchronization in the ECG waves. With the present advancement in technology, there is a great scope for developing robust medical ECG devices in analyzing the ECG signals and classify the patient condition. CVD classification using Phase Space Reconstruction (PSR) based techniques^2,3^ was proposed to potentially impact the diagnosis of Ventricular Arrhythmias (VA), it is also useful in the context of indexing life-threatening conditions leading to sudden coronary death. PSR is also widely used in the field of nonlinear dynamics to detect the minute desynchronization in time-series data^4,5^. Literature^6^ has used the PSR technique for the automatic speech recognition, whereas the literature^7^ focused on the review of nonlinear dynamic system analysis for the classification of ECG signals. Hence, PSR technique will have the potential in notifying even a small change in the localized features (PR interval, QRS complex, and QT interval) of the ECG wave, which are leading to CVD. Few investigations^8–12^ have shown that the PSR technique has the ability to represent ECG time series data into a 2D-image format known as phase portrait to identify the indexes of QRS complex^13,14^ and precisely detect the desynchronization in the ECG waves. The phase portrait is obtained by plotting the original ECG signal with respect to its delayed version on the 2D plane. The trajectory of phase portrait for the healthy control looks like a clean and closed contour as opposed to the diseased cases where the contour will be chaotic in nature. Therefore, observing and processing these portraits could lead to the classification of CVD.

The detection and classification of VA, based on the aforementioned PSR technique, were proved to happen accurately^3^. However, the existing PSR technique is not generic and cannot be extended to many of the CVD (Atrial Fibrillation (AF), Bundle Branch Block (BBB), Myocardial Infarction, Cardiomyopathy, Dysrhythmia, Hypertrophy) related to irregular P waves, fragmented QRS complex, ST elevation/depression or any desynchronization in the localized features. The shortcomings of using the entire ECG frame reported in the literature over the localized features are listed as follows

- Accuracy of the classification will decrease by considering the entire ECG frame compared with the localized features.
- b. Detecting the type of CVD (Atrial Fibrillation (AF), Bundle Branch Block (BBB), Myocardial Infarction (MI), Cardiomyopathy (CY), Dysrhythmia (DY), Hypertrophy(HY)) will be difficult if the entire ECG frame is considered.
- Predicting the departure from the healthy condition to unhealthy condition corresponding to the CVD (AF, BBB, MI, CY, DY, HY) will be easy by using the localized features.

Motivated by this, here we have introduced the concept of localized features to mitigate all the aforementioned limitations. These are the reasons for which the ECG frame based classification as per the reported literature have the shortcomings that is rectified in the proposed method. It is evident from the above statement that all the aforementioned diseases can be detected using the localized diagnostic features that perhaps would be given a miss if the entire ECG frame is considered. Therefore, to mitigate the above limitations our attempt here is to propose a generalized PSR based detection and classification of the CVD by exploiting the localized features of the ECG unlike the state-of-art PSR techniques^8–12^. One of our published paper^15^ introduced our preliminary idea of using the localized features (PR interval and QRS complex) for the detection of abnormalities in the ECG wave. For the first time, we have exploited the usage of localized features with the PSR technique to detect the abnormalities. However, we have taken only PR interval and QRS complex as a part of preliminary study and able to detect the abnormalities based on the box-count distribution. This has motivated us to do the proposed work where we achieved the overwhelming results as reported in this manuscript. In this proposed work, we are extrapolating the idea further by incorporating another important feature ‘QT interval’ to detect other CVD and propose a generic methodology. We have performed in-depth analysis by setting up the real-time continuous ECG data and performing PSR technique on continuous healthy and unhealthy ECG waves. Coefficient variation of all the cases are calculated using the sliding window which is holding the box-count distribution values. Based on achieved statistical analysis (ANOVA, Confidence Interval) and diagnosis measures, we conclude that the proposed methodology can be extended to classify most of the CVD cases.

To the best of our knowledge, this is the first of its kind work which is elaborated in this article with a detailed discussion and substantiated by rigorous results and analysis.

## Results and Discussion

**Figure 1:**
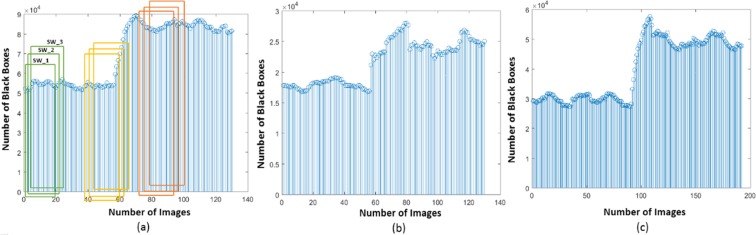
Box-count distribution of PSR images, case (**a**–**c**) corresponds to QT interval, PR interval and QRS complex respectively. *SW: Sliding Window (Box-count values of 20 PSR images), Green Window: Healthy QT interval, Yellow window: Healthy + Unhealthy QT interval, Orange: Unhealthy QT interval.

**Figure 2:**
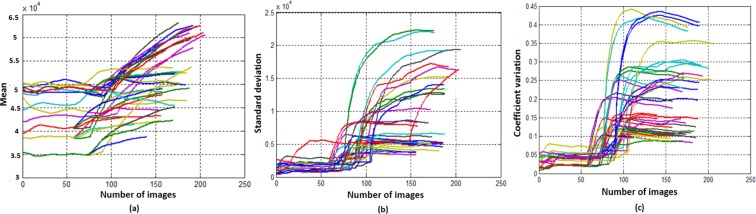
Plots of statistical measurements. (case **a**) Mean distribution of box-counts for healthy followed by unhealthy QT intervals, (case **b**) SD distribution of box-counts for healthy followed by unhealthy QT intervals, (case **c**) CV distribution of box-counts for healthy followed by unhealthy QT intervals.

The performance of the proposed methodology has been tested using the PTBDB and IAFDB databases^16^. Healthy ECG samples followed by unhealthy samples of these databases are taken as different arrays (ECG database). The outcome from the FE block of all these arrays give the localized features, applying PSR technique on these features results in PSR images, Fig. 1 shows the distribution of box-count in each image corresponding to the localized features. To classify the ECG signals, the mean, Standard Deviation (SD) and the coefficient of variation are calculated on these box-count plots using the sliding window technique, where the window occupies 20 consecutive box-count values for the analysis. When the window move stepwise towards right, it occupies previous 19 box count values as shown in the Fig. 1(a). Initially, the window (Green color) occupies box-count values corresponding to healthy QT intervals, as the window moves stepwise towards right it occupies the box-count values of partial unhealthy (Orange color window) QT intervals, moving further the window occupies only unhealthy box-count values (Red color window) as shown in Fig. 1(a). Mean, SD and CV are calculated for each window of QT interval array and plotted the values as shown in Fig. 2.

From the Fig. 2, it is clear that the mean and SD are almost consistent over the ${\mathscr{X}}$-axis until the occurrence of the abnormal box-count values (corresponding to orange color window in Fig. 1(a)). With the beginning of unhealthy features, we can observe an abrupt change in the box-count which results in the sharp increase of mean and SD as shown in the Fig. 2(a,b), this indicates a gradual increment of desynchronization in the localized features. The windows corresponding to healthy PSR images may not have the same mean and SD values as shown in Fig. 2(a,b), we can observe minute variation in the values based on the trajectories spread over the image. Similarly, for the unhealthy intervals, the mean and the SD values may not be the same and vary based on the image. Hence, it is difficult to assign a threshold value for the mean and SD to classify the normal and abnormal signals. Therefore, another approach to knowing the spread of the trajectories is to calculate the CV for all the windows to classify the ECG signal, the CV trends of the QT interval is shown in Fig. 2(c).

**Figure 3:**
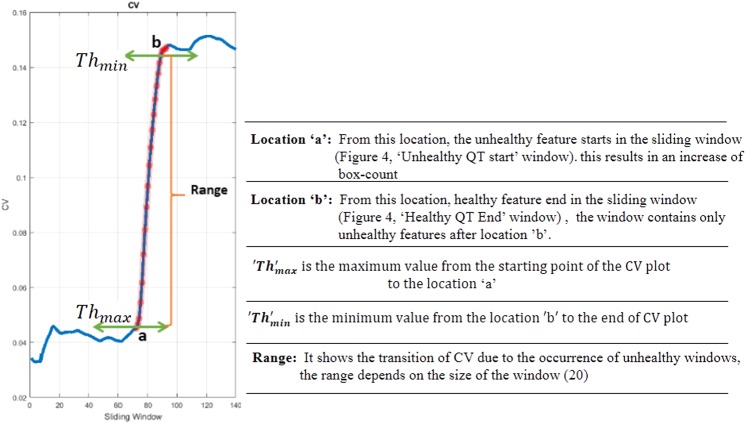
Coefficient Variation of single patient.

From visual inspection, CV plots of all the patients in Fig. 2(c) maintain ‘almost constant value till the occurrence of unhealthy QT interval, the plots follow an inclination due to the transition from healthy to unhealthy QT intervals. The CV trends motivate us for fixing two thresholds for the classification of ECG signals. The reason for choosing two thresholds is explained by considering the CV plot of a single patient as shown in Fig. 3. The horizontal lines on the image marked at two points are ‘a’ and ‘b’, the CV value at the point ‘a’ defines the beginning of the unhealthy interval and the value at the point ‘b’ defines the end of healthy interval of the window (shown in the Figure having caption “QT intervals (healthy followed by unhealthy) with sliding window” from the Methods section) with the window names ‘Unhealthy QT start’ and ‘Healthy QT end’ respectively. From the CV plot shown in Fig. 3, the starting value of the CV till the point ‘a’ gives the CV values for the healthy windows, whereas from the point ‘b’ till the endpoint of CV indicates the CV values of unhealthy windows. The number of values between the points ‘a’ and ‘b’ defines the range. The size of the range depends on the window size, in our work we have chosen the window size as 20. The two threshold values are formulated by taking the maximum value (Thmax) in the CV plot from the starting value of the CV till ‘*a*’ (Fig. 3) and the minimum value (Thmin) from ‘*b*’ (Fig. 3) to the end of the CV plot respectively. Likewise, we have noted down the maximum and minimum values from all the CV plots corresponding to the PR interval, QRS complex, and the QT interval.

The thresholds for the classification have been fixed by taking the maximum (Th_final_max_) from all the maximum CV values and the minimum (Th_final_min_) from all the minimum CV values of all the patients as described in Eqs (1) and (2). The CV threshold (CVth = (Th_final_max_ = 0.079, Th_final_min_ = 0.082)) of QT interval are shown in Table 1.

1 $$T{h}_{final\_\max }=Max(T{h}_{\max 1},T{h}_{\max 2},T{h}_{\max 3}\mathrm{........}T{h}_{\max 65})$$

2 $$T{h}_{final\_\min }=Min(T{h}_{\min 1},T{h}_{\min 2},T{h}_{\min 3}\mathrm{........}T{h}_{\min 65})$$

**Table 1:** Performance results of proposed methodology on localized features.

Parameters	PR Interval (N = 65)			QRS Complex (N = 65)			QT Interval (N = 65)		
	H	UnH	UnH-H	H	UnH	UnH-H	H	UnH	UnH-H
CVth	0.068	0.1012	0.033	0.069	0.083	0.011	0.079	0.082	0.003
Mean	0.037	0.171	0.133	0.049	0.217	0.168	0.047	0.180	0.133
SD	0.0135	0.049	0.046	0.01	0.077	0.073	0.013	0.175	0.179
95% LCL	0.034	0.158	0.121	0.046	0.198	0.15	0.043	0.136	0.177
95% UCL	0.041	0.183	0.144	0.051	0.236	0.186	0.050	0.223	0.088
Mean CV Difference of UnH and H (95% CI)	(0.12195, 0.14435)			(0.151705, 0.185705)			(0.09004, 0.176044)		
t-critical	1.669			1.669			1.669		
t-statistic	23.32			18.62			5.96		
p-value	2.71 × 10^−33^			7.85 × 10^−27^			5.88 × 10^−8^		
F-statistic	541.69			346.74			35.542		
Ability of classification	—	—	Yes	—	—	Yes	—	—	Yes

*Healthy = H, Unhealthy = UnH.

If the CV value of the patient lies below or equal to the maximum threshold (Th_final_max_), then the patient is classified as normal, if the CV value crosses the minimum threshold (Th_final_min_) value then the patient is said to have the abnormal condition (Eq. (3)). We have observed that, if the CV value lies between the maximum and the minimum threshold values, then the patient is going to be abnormal state and need medical emergency, this observation leads to the predictive analysis in classifying the ECG abnormalities in a proactive way.

3 $$\begin{array}{l}CV\le T{h}_{final\_\max }(normalcondition),CV\ge T{h}_{final\_\min }(Abnormalcondition),\\ \,\,\,\,\,\,\,\,T{h}_{final\_\max }\ge CV\le T{h}_{final\_\min }normaltoabnormalstate\end{array}$$

**Figure 4:**
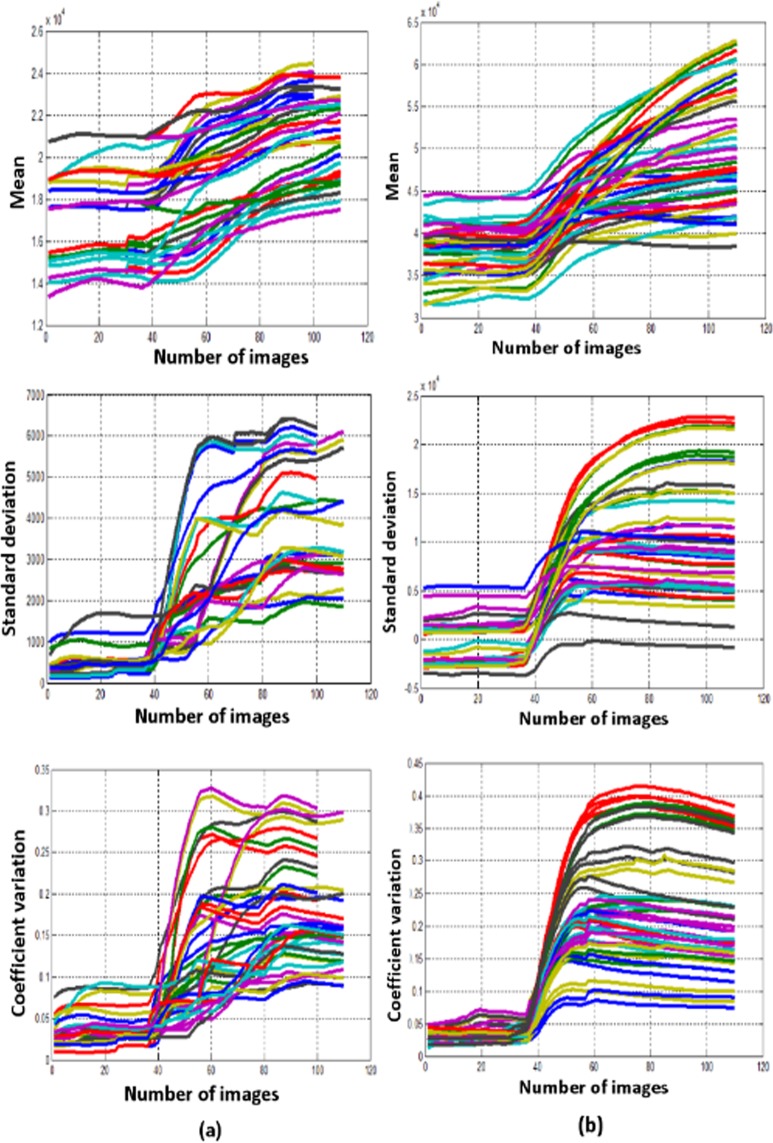
Plots of statistical measurements. (case **a**) Mean, SD and CV of box-counts corresponding to the PR intervals. (case **b**) Mean, SD and CV of box-counts corresponding to the QRS complexes.

The mean, SD, and CV plots of PR and QRS complex arrays are shown in Fig. 4. The similar procedure is followed for PR interval and QRS complex arrays to find the coefficient variation of box-count distribution (Fig. 1(b,c)), threshold values (T_final_max_ and T_final_min_) are calculated based on Eqs (1) and (2). Table 1 shows the mean, SD and threshold values of coefficient variation (CV_th_) of PR interval and QRS complex to define the condition of the patient. It is observed that, if the value of CV plot corresponding to PR interval and QRS complex are below the threshold 0.068 (T_final_max_PR_), 0.069 (T_final_max_QRS_) respectively then the patient is said to be in normal condition, else, if the values are greater than or equal to the threshold 0.1012 (T_final_min_PR_), 0.083 (T_final_min_QRS_) respectively then abnormal condition is detected in the corresponding localized features. Whereas, if the CV values are between these ranges then the patient is said to tend towards the abnormal condition from normal condition, this helps the patient or the caretaker to adopt a proactive measure. To know whether the proposed methodology is suitable for the clinical relevance, we have calculated the confidence intervals, the p-value (probability), and ANOVA test to the coefficient variations of all the localized features^17^.

### Classification, confidence intervals and ANOVA analysis

Confidence intervals provide a range in which the true value lies with a certain degree of probability. For the statistical analysis, we usually select confidence levels of 95%^17^, the values of Upper Confidence Level (UCL) and Lower Confidence Level (LCL) of all the localized features are shown in the Table 1, Upon observation, it is noticed that the UCL of healthy cases in all the localized features is always less than the LCL of unhealthy which says that the obtained CV value for the healthy cases will not cross the LCL of unhealthy cases. Considering the QT interval case, the difference in mean CV is between 0.09004 and 0.176044 with unhealthy having the higher values. Since the obtained mean difference values of all the localized features do not include the null value (zero) as shown in Table 1, we conclude that there is a significant difference in mean CV between healthy and unhealthy cases.

For more evidence of statistical significance, we have performed the repeated-measure Analysis of Variance (ANOVA)^17–21^ and calculated the p-value for all the localized features. Based on the subjects (65 patients) tested, the degrees of freedom between (df between = 1) and degrees of freedom error (df error = 64) are calculated to find the critical value (3.991) from the F-table. The decision rule is taken such that the null hypothesis is rejected if the obtained F- statistic value is greater than the critical value. The ratio of the mean square between and mean square error are used to calculate the F-statistic value. The obtained F-statistic values for all the localized features are shown in Table 1, it is clear that the calculated values are far greater than the critical values and we conclude that the mean of healthy and unhealthy differed significantly.

4 $$\begin{array}{rcl}T{h}_{mean\_max} & = & mean(T{h}_{max1},T{h}_{max2},T{h}_{max3}\mathrm{...}T{h}_{max65})\\ T{h}_{mean\_min} & = & mean(T{h}_{min1},T{h}_{min2},T{h}_{min3}\mathrm{...}T{h}_{min65})\end{array}$$

While calculating the p-value, we are testing whether the mean CV of unhealthy is greater than the mean CV of healthy cases, so this has to be alternative hypothesis and the condition will be (*Th*_mean_min_ > *Th*_mean_max_), and for the null-hypothesis, the condition will be (*Th*_mean_min_ ≤ *Th*_mean_max_)). Using the Eq. (4), *Th*_mean_min_ *and Th*_mean_max_ are calculated. The p-value reflects the measure of strong evidence against the null hypothesis, for the critical or the rejection the significance level *α* = 0.05 is chosen to calculate the p-value. Since this is a one-tailed test we use the one tailed critical value here and because it is a right tailed test we reject the null hypothesis if the obtained t-statistic value is greater than the critical value. Considering the QT interval case shown in Table 1, the obtained test-statistic value (5.96) is greater than the critical value (1.669), as a result we will reject the null hypothesis. P-value is also used to arrive at the same decision, the p-value role is to reject the null hypothesis if the obtained p-value is less than alpha, because this is a one-tailed test a p-value would be a one tailed p-value (5.88 × 10^−8^), which is less than alpha of 0.05 and that’s again tells us to reject the null hypothesis. The similar conditions (Alternative, Null hypothesis and alpha value) are taken for the PR interval and QRS complex to find the t-statistic and p-value, the obtained values are shown in Table 1, it is clear that the t-statistic values are greater than the critical values and the p-value is less than 0.05. Our conclusion will be that there is enough evidence to infer that the mean CV of unhealthy cases is greater than the mean CV of healthy cases for all the localized features.

The proposed methodology has been found to have higher sensitivity and specificity values as shown in Table 2. In a statistical sense, the false positive detection is found to be zero in our analysis of 65 subjects, the diagnosis measures like sensitivity (Se) or true positive rate (TPR), specificity (Sp) or true negative rate (TNR), accuracy (Acc), precision or positive predictive value (PPV), negative predictive value (NPV), fall out or false positive rate (FPR), false discovery rate (FDR), miss rate or false negative rate (FNR) and F_1 score are calculated^22^ using the Eq. (5) and the values are shown in Table 2. The aforementioned studies show that the proposed PSR methodology helps in detecting the chaotic behavior and classify the ECG abnormalities using the localized features.

5 $$\begin{array}{l}Se=TP/P=TP/(TP+FN),Sp=TN/N=TN/(FP+TN),Acc=(TP+TN)/(P+N),\\ PPV=TP/(TP+FP),NPV=TN/(TN+FN),FPR=FP/N=FP/(FP+TN),\\ FDR=FP/(FP+TP)=1-PPV,FNR=FN/(FN+TP),{F}_{1}score=2TP/(2TP+FP+FN)\end{array}$$

**Table 2:** Diagnosis measures of proposed methodology on localized features.

Diagnosis measures (%)	PR Interval	QRS Complex	QT Interval
Sensitivity or TPR	95.38	96.923	98.46
Specificity or TNR	100.00	100.00	100.00
Accuracy	97.69	98.46	99.23
Precision or PPV	100.00	100.00	100.00
NPV	95.58	97.01	98.48
FPR	0.00	0.00	0.00
FDR	0.00	0.00	0.00
FNR	100.00	100.00	100.00
F_1 score	97.63	98.43	99.22

In Table 3 we have compared the proposed methodology performance with other methods. It can be noted that, other methods have considered whole ECG beat for the classification and the accuracy levels of all these references have been measured by observing the number of failed cases to the total number of cases taken as input. Since we are introducing for the first time the localized features based methodology, therefore we have adopted the similar procedure to find the accuracy of the proposed methodology. Considering the localized feature QT interval, we have taken 65 cases for the validation, out of which the healthy and unhealthy Coefficient of Variation (CV) of 64 cases are satisfying the CV thresholds values of healthy and unhealthy respectively as shown in the Table 1. For one test case out of 65 cases, the obtained CV values are not in the expected threshold range which resulted in 98.5% accuracy, this is due to the presence of lot of noise in that particular case, PSR yields best results if the signals are noise-free. Similarly, for the PR and QRS complex we have taken each of 65 cases to validate the proposed methodology, out of which 62 and 63 cases are within the CV thresholds which resulted in 95.3%, 96.9% accuracy respectively.

**Table 3:** Performance comparison with the existing methods.

Type of CVD	Database	Classifier	Classification performance
MI	21 New Zealand rabbits	KNN, SVM^29^	Accuracy = 98.6, 93.5%
MI	MCG data	Maximum current density approach^30^	Sens = 91.2%, Spec = 84.6%
MI	PTBDB	CNN^31^	Accuracy = 96%
MI	PTBDB	Harmonic phase values^32^	Accuracy = 95%
BBB	UHSF	SWT^33^	Accuracy = 94.1%
BBB	MIT-BIH	Genetic algorithm neural network^34^	Accuracy = 98%
BBB	MIT-BIH	Random forest based classification^35^	Accuracy = 98.4%
AF, BBB, MI, CY, DY, HY	PTBDB	PSR (Proposed method)	Accuracy = 95.3, 96.9, 98.5%

*MCG = (magnetocardiography), UHSF = University Hospital Southampton NHS Foundation.

**Figure 5:**
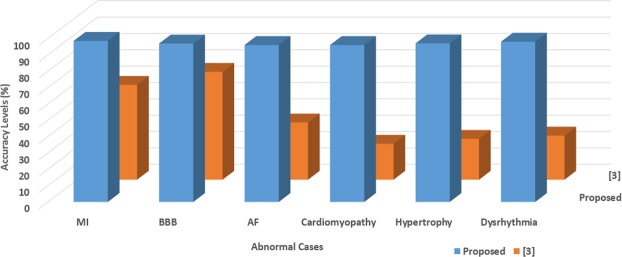
Comparison of accuracy by the proposed methodology and existing work.

The outcome of the proposed methodology is thoroughly analyzed on all the databases and compared with the result of the statistical trends (distribution of box count values) of^3^, the accuracy values compared are shown in the Fig. 5 and and Table 3. The accuracy of the MI and BBB abnormal cases is higher compared to other cases, this is due to more variation in the QT and QRS complex. Since the existing work^3^ take the complete beat in the window, the minute change in the localized feature is not reflecting the high variation in the box-count of the PSR plot, hence the proposed methodology is achieving more accuracy. Based on the statistical outcome and the comparison with the literature we have enough evidence to conclude that the PSR technique on the localized features shows medical significance in classifying the ECG abnormalities.

Since our aim is to do the real-time ECG classification on an edge-device that is running under resource constrained environment with scarcity of power and area, therefore our idea here is to propose a low-complexity yet accurate solution and therefore we have adopted classical technique of localized features detection followed by three class classifier. Therefore, we do not propose to use here SVM, CNN or KNN based computational intensive methodology that may result in increasing the overall hardware complexity of the edge device. If the learning based algorithms such as SVM, KNN or CNN are used or designed that also can be trained on the localized features that we are introducing here in this paper. However, applying SVM, KNN or CNN on localized features for the classification would be future scope of work. The proposed localized features based detection and hypothesis testing and simplistic classification (the one proposed here) would help translate the proposed methodology into a real-life edge device.

**Figure 6:**
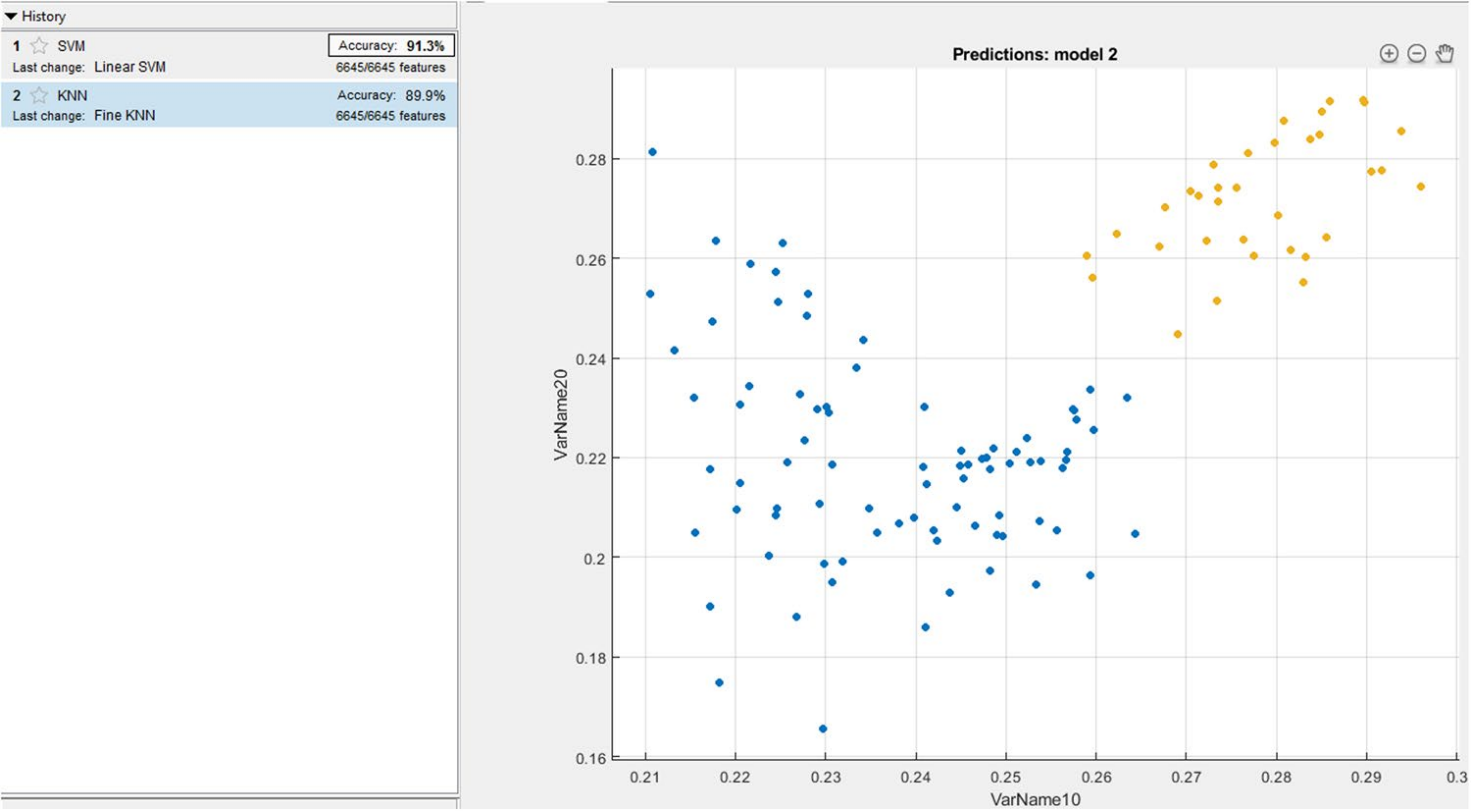
SVM and KNN analysis on PR interval.

**Figure 7:**
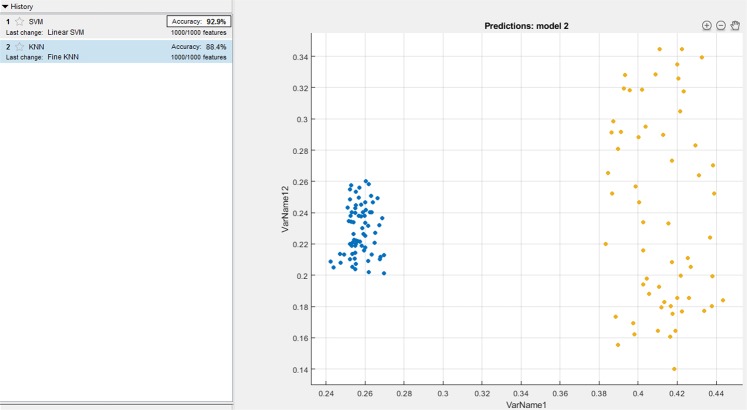
SVM and KNN analysis on QRS complex.

**Figure 8:**
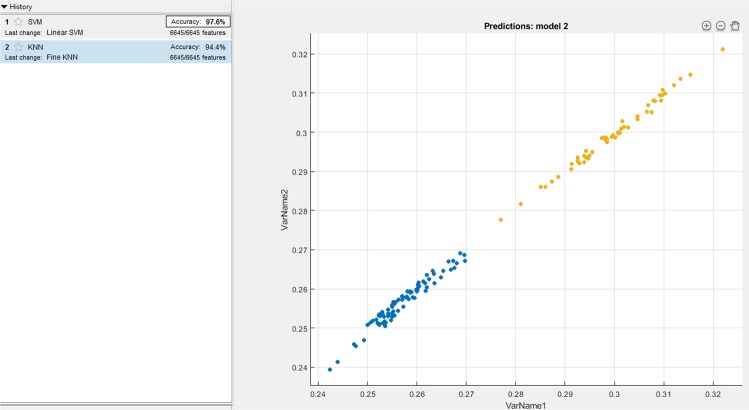
SVM and KNN analysis on QT interval.

**Figure 9:**
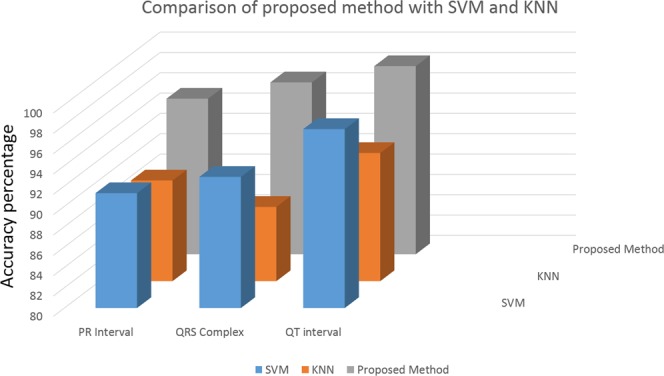
Comparison analysis of SVM, KNN and proposed PSR method.

Since KNN and SVM based learning algorithms have been widely used for classifications in the domain of signal processing, authors have compared the proposed method with the existing SVM and KNN based classifiers for the bench marking analysis. We have performed SVM and KNN techniques on the localized features (PR interval, QRS complex, and QT interval) of all the 65 patients. For the analysis, 5 fold cross-validation is performed on the localized features for the training and testing. Figures 6, 7 and 8 shows the accuracy values achieved with SVM (91.3%, 92.9%, 97.6%) and KNN (89.9%, 87.3%, 92.6%) based classifier for the PR interval, QRS complex and QT interval respectively. Authors here perform a bench marking analysis of the proposed PSR based classifier with the SVM and KNN accuracy results, the comparison results are shown in the Fig. 9. From the bar graph shown, we can observe that SVM, KNN and the proposed methods are applied on the individual diagnostic features viz. PR, QRS and QT interval and the accuracy levels are shown, the accuracy of the proposed method performs better than SVM by 4%, 4%, 0.9% respectively. Similarly, proposed method performs better than KNN by 5.4%, 9.6%, 5.7% respectively in terms of accuracy.

## Methods

**Figure 10:**
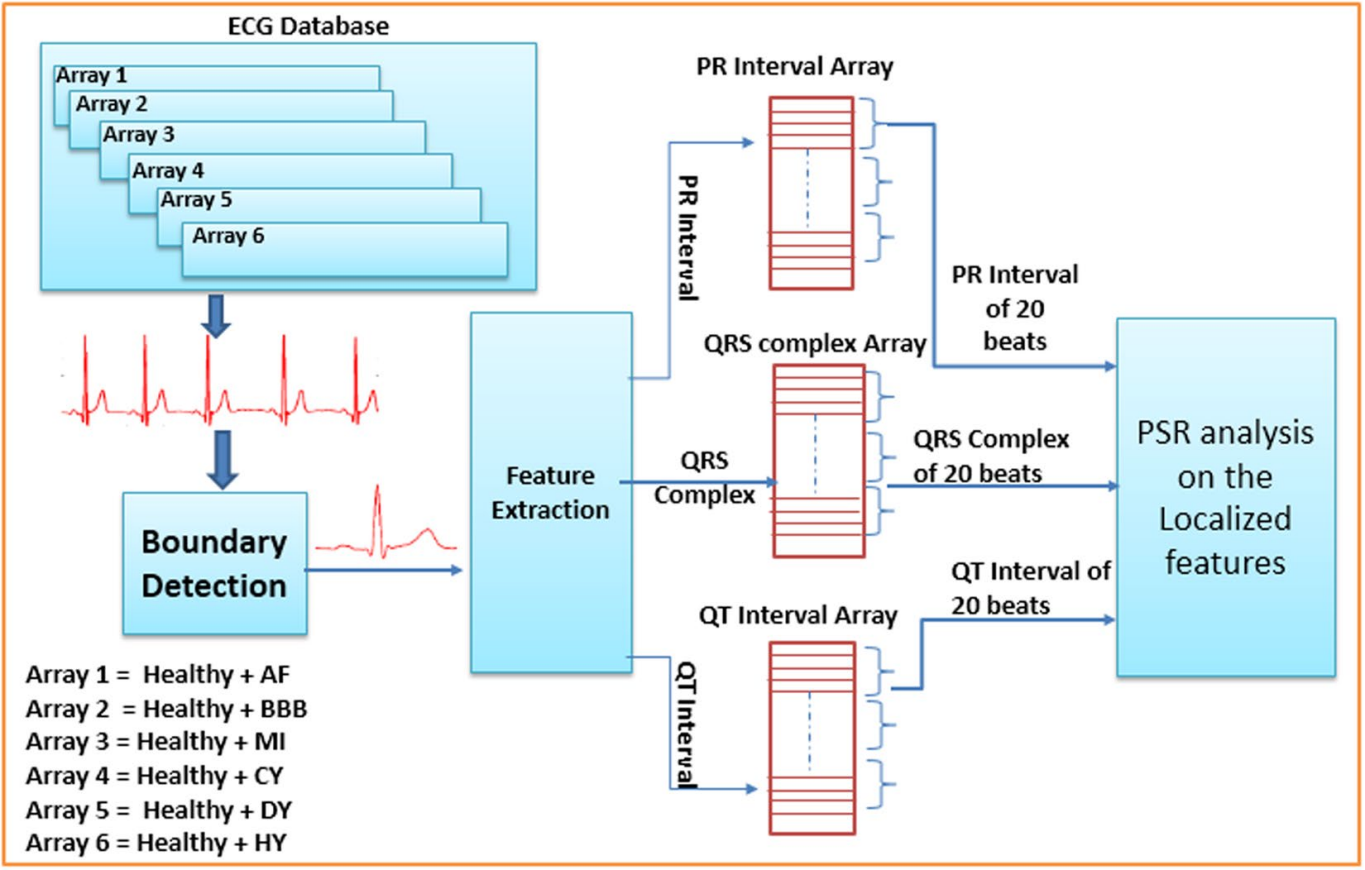
Block level diagram of the proposed methodology.

**Figure 11:**
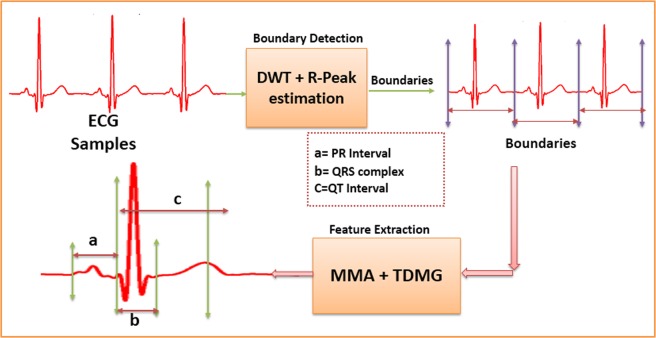
Extraction of boundaries and localized features of continuous ECG signal.

For the present work, the ECG databases have been taken from publically available Physionet^16^, to classify healthy and unhealthy conditions 65 cases were selected from the Physikalisch-Technische Bundesanstalt diagnostic database (PTBDB)^23^ and Intracardiac Atrial Fibrillation Database (IAFDB)^24^, which are sampled at 1 KHz. Healthy ECG samples followed by unhealthy samples (Atrial Fibrillation, Bundle Branch Block, Myocardial Infarction, Cardiomyopathy, Dysrhythmia, Hypertrophy) of these databases are taken as different arrays (ECG database) as shown in Fig. 10. The boundaries of each ECG beat from the continuous ECG wave of each array are extracted using our proposed Boundary Detection (BD) block^25^ as shown in Fig. 11, making use of these start and end boundary indexes of each ECG beat we have extracted the localized features (PR Interval, QRS complex, and QT interval) using our proposed Feature Extraction (FE) block^25,26^. The proposed work is feature-based classification methodology where all the localized features (PR interval, QRS complex, and QT interval) are accessed or extracted using the method proposed in our earlier work^25^. Authors have contributed in the domain of accurate online features extractions that had been evaluated against the widely accepted publically available database (PTBDB, MIT-DB, IAFDB, CSEDB). Since that has been verified with the CSE standard and it also has been tested and validated by the doctors; therefore, it can be noted that all the features mentioned in this manuscript are not only limited to 65 patients and the feature extraction method is generic with mathematical insights. Both the blocks (BD and FE) use the common Haar Discrete Wavelet Transform (DWT) to extract the coefficients of the ECG signal, third level Haar coefficients are used to extract the QRS complex and boundaries of each beat, whereas fifth level Haar coefficients are used to extract the PR and QT interval as shown in Fig. 11, FE block is the combination of Modulus-Maxima Analysis (MMA) and Time Domain Morphology (TDMG)^26^. The Individual features PR Interval, QRS complex and QT interval of each beat are stacked in their respective arrays (PR interval array, QRS complex array, and QT interval array) as shown in Fig. 10. PSR technique is performed to detect and classify abnormalities in these localized features.

To achieve accurate results for the proposed methodology, ECG signals were filtered to remove the noise using Butterworth high-pass digital filter with a cut-off frequency of 1 Hz and fed the ECG signal to the low-pass filter with a cut-off frequency of 40 Hz to remove the noise and the baseline wandering^27^. The filtered ECG signal is normalized using the Eq. (6) such that all the values will be in the range of ‘0’ and ‘1’^28^.

6 $${E}_{n}(t)=(E(t)-{E}_{min}(t))/({E}_{max}(t)-{E}_{min}(t))$$

where *E*_*n*_(*t*) is the normalized ECG signal, *E*_*max*_ (*t*) and *E*_*min*_ (*t*) are the maximum and the minimum values of the ECG signal *E*(*t*).

The vision of this present study is to classify various cardiovascular diseases by introducing a method based on localized features. For the first time, such localized features have been used for the domain of CVD classification with the help of statistical parameters (mean, standard deviation and coefficient variation) as shown in Table 1. We have developed our classification method and computed these statistical parameters as follows: out of 65 patients, we have taken the abnormal cases (AF, BBB, MI, CY, DY, HY) and sectioned into 3 (PR interval, QRS complex, and QT interval) classes. For all these classes, we have developed the proposed model by taking 80% of the ECG data, applying the fore-mentioned features extraction methodology and computed the statistical parameters. Remaining 20% data had been used for validation of the proposed localized feature based classification algorithm.

### Phase space reconstruction and box counting

**Figure 12:**
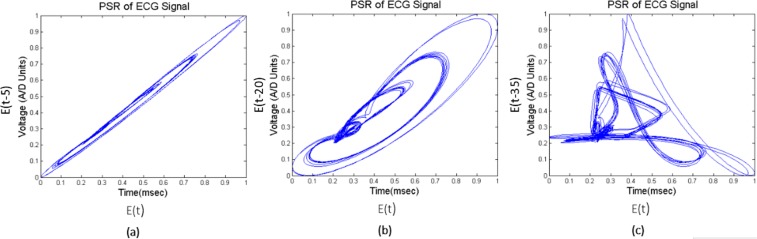
PSR plots with different delay time units.

PSR is widely used in the field of nonlinear dynamic systems to detect even the minute difference in time-series data^1,2^ since ECG arrhythmias also behave the similar chaotic nature which made us apply the PSR technique to the proposed method. We add the delay (‘T’) to the time series ECG data *E*_n_(*t*), where the delay value (20 msec) is selected statistically, such that phase space trajectories have the maximum span by plotting the original signal and the delayed signal^3^ to generate the PSR image. In this work we have taken three delay values viz. 5 msec, 20 msec and 35 msec. The comparison between them is interpreted by plotting three PSR images using these delays for the same ECG wave as shown in the Fig. 12. In Fig. 12(a), the time delay is set to be 5 msec, the resulting trajectories in the phase space domain take place on the diagonal axis since the consecutive values which are plotting are similar, this leads to the suppression of features. In Fig. 12(b), the time delay is chosen to 20 msec, the phase portrait of the signal is clearly distributed over the ${\mathscr{X}}$ and ${\mathscr{Y}}$ axis, we can also notice that the outer trajectories corresponding to the QRS complex and inner small trajectories represent P and T waves respectively. In the Fig. 12(c), the time delay is set to 35 msec. Here, we can notice that the excess time delay leads to the phase portraits overlap each other and to be disjointed by over stretching and leads to complicated internal graphs. If the delay is too small or too high, the reconstructed phase plot is very close to the diagonal line or spread in the phase space respectively. Hence 5 msec or 35 msec lead to the misclassification of ECG. We have followed the procedure of taking the optimum delay time mentioned in the book^5^. Here our main idea is to propose the methodology and we have proved that high amount of accuracy is achieved when we have taken 20 msec time delay.

**Figure 13:**
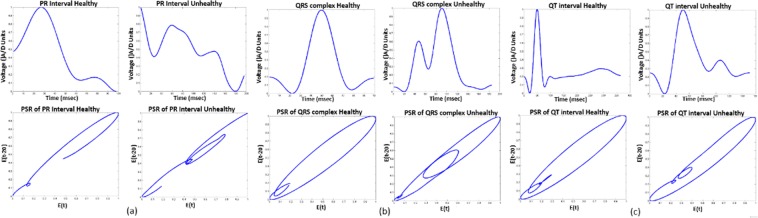
PSR images of PR interval, QRS complex and QT interval with healthy and unhealthy cases.

We can observe the phase space trajectories and the amount of spread in the image using the well-known technique of box-counting^6^, to analyze the statistical behavior of the ECG signal. The 2D image (Fig. 13(a)) is a phase space diagram of N × N pixels, where ‘N’ is an integer. The trajectories in the image pass through the pixels are considered as black boxes (nb), and the rest of the pixels are considered as white boxes (nw). If the classification is performed using the PSR technique, then the box-count calculation will be the basic step for understanding the statistical variation of trajectories in the PSR image to perform the classification analysis. The box-count is used in the literature^3^ in this domain hence we have adopted the same concept and focused to enhance the existing work such that it can cover various CVD classification. The concept of using the box-count does not limit the novelty of the proposed methodology.

The chaotic nature of ECG arrhythmia results in higher box count of black boxes than the white due to the spread of trajectories compared to the healthy ECG signals^15^. Figure 13(a) shows the image of the healthy, unhealthy PR interval wave and its corresponding PSR plots, observing both the PSR plots of healthy and unhealthy we can interpret that the unhealthy PSR plot has spread more trajectories passing through many pixels and results in an increase of the box count in the PSR image than the healthy PR interval. Similarly, Fig. 13(b,c) shows the healthy and unhealthy QRS complex, QT interval and its PSR plots respectively. The number of boxes in the PSR plot of healthy and unhealthy will differ based on the irregularities in the wave. Thus we can see the increment of box-count due to the chaotic nature of localized features^15^ for unhealthy cases. In this paper, to detect and classify the abnormalities in the localized features of continuous ECG wave we took the combination of healthy and unhealthy individual localized features and stacked as arrays shown in Fig. 10. The classification procedure on all the localized features is performed in a similar fashion, for the explanation we have demonstrated the proposed method on the QT interval array in the below subsection.

### Analysis of the proposed PSR technique on localized features intervals

#### An example of the methods based on QT interval

**Figure 14:**
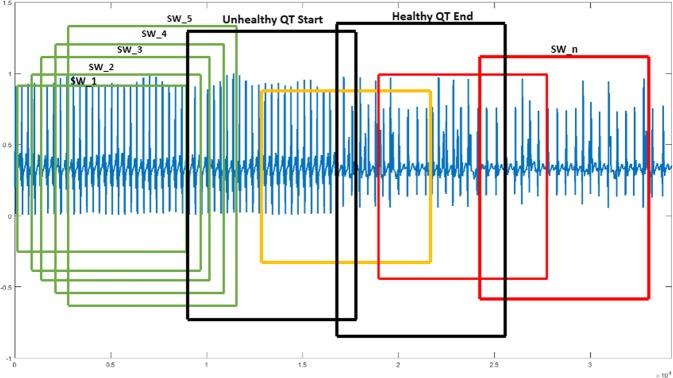
QT intervals (healthy followed by unhealthy) with sliding window. *SW: Sliding Window (Occupying 20 QT intervals), Green Window: Healthy QT interval, orange window: Healthy + Unhealthy QT intetrval, Red: Unhealthy QT interval.

The start (QRS_on_) and end index (T_off_) of QT interval of each ECG beat from the FE block are stacked in the QT interval array as shown in the Fig. 10. QT interval of each ECG beat is known to us from the FE outcome and can be represented as shown in the Eq. (7), where QT(i) is the i_th_ QT interval. Considering, the array is having the healthy QT interval samples followed by unhealthy QT interval samples as shown in the Fig. 14. The windowing technique is applied on the QT interval array such that the window occupies 20 consecutive QT intervals as described in Eq. (8), for every step the window moves towards the right (SW_2) and overlap 19 QT intervals with the previous window (SW_1) as shown in the Fig. 14. In general, if the sliding window holds ‘W_1_’ number of QT intervals and ‘W_2_’ phase portrait images, then the nth phase portrait represents the phase-space behavior from nth to (n + W_1_ − 1) number of consecutive QT intervals. If we consider W_1_ = 20 and W_2_ = 90 then the number of QT intervals covered to find the statistics are (W_1_ + W_2_ − 1) = 109 consecutive QT intervals.

**Figure 15:**
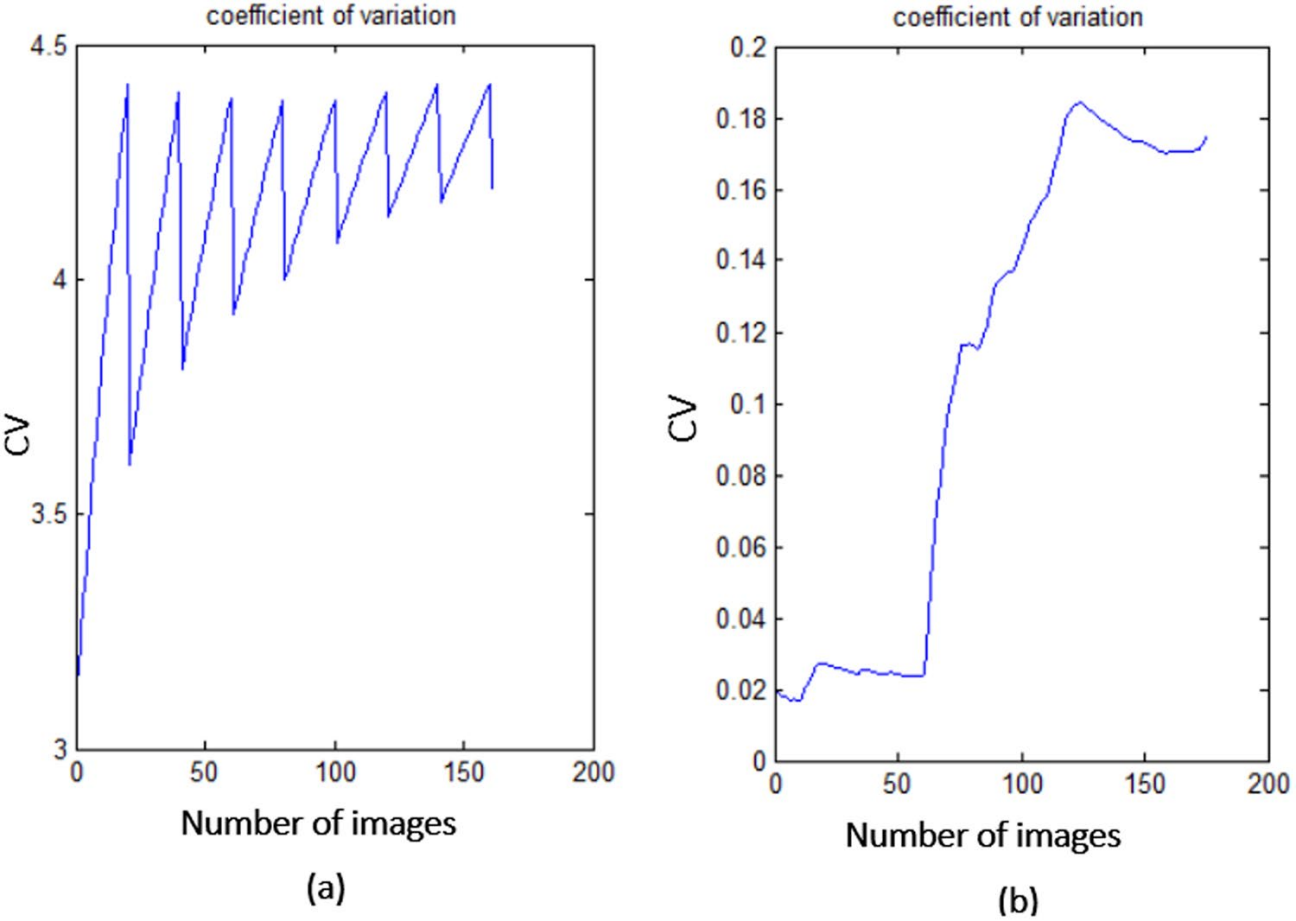
CV plots obtained using static and sliding window respectively.

In the manuscript, sliding window technique is applied in two cases. In the first case, it used to occupy the box-count distribution graph for statistical analysis of PSR images (Fig. 1). It is also employed on the continuous localized features for plotting the PSR images (Fig. 14) as the second case. In the continuous waveforms, each and every feature of the waves are correlated, the necessity of using the sliding window and the moving step by ‘1’ is to observe the correlation of the features such that we may not miss them and it also helps to track the trending towards the abnormality. For example, if we take a static window instead of sliding, then the static window occupies the first 20 consecutive waveforms/box-count graphs and the statistical parameters are calculated, when the window moves stepwise towards right, the static window occupies the waveforms from 21 to 40 and the statistical parameters are calculated. Upon observation with the static window or higher moving step, we can say that the run-time statistical parameters (mean, SD, CV) will miss the features correlation from the waveform 2 to 39 and leaving the chaotic motions unnoticed if there are any healthy to unhealthy transformations between 2 to 39. Figure 15(a,b) shows the CV plot using the static and sliding window respectively, the CV obtained using the static window is random and will be difficult to assign a threshold value for the classification. Hence, the above explanation justifies the usage of sliding window and this motivates us to use it for the classification purpose. The length of the window is kept higher to capture the different statistical moments. The histogram of the number of box count (Fig. 1) scanned by the sliding window needs to be constructed in sufficient details, which requires large number of data points corresponding to localized features.

7 $$Q{T}_{{\rm{interval}}}=QT(i),\,i\in \{\mathrm{1,}\,\mathrm{2,}\,\mathrm{3,}\,\mathrm{...,}\,(n+\mathrm{1)}\}$$

8 $$[\begin{array}{llll}QT\mathrm{(1)} & QT\mathrm{(2)} & QT\mathrm{(3)......} & QT\mathrm{(20)}\\ QT\mathrm{(2)} & QT\mathrm{(3)} & QT\mathrm{(4)......} & QT\mathrm{(21)}\\ QT\mathrm{(3)} & QT\mathrm{(4)} & QT\mathrm{(5)......} & QT\mathrm{(22)}\\ \mathrm{.} & \mathrm{.} & \mathrm{.} & \mathrm{.}\\ \mathrm{.} & \mathrm{.} & \mathrm{.} & \mathrm{.}\\ \mathrm{.} & \mathrm{.} & \mathrm{.} & \mathrm{.}\\ QT(n-\mathrm{19)} & QT(n-\mathrm{18)} & QT(n-\mathrm{17)......} & QT(n)\end{array}]$$

**Figure 16:**
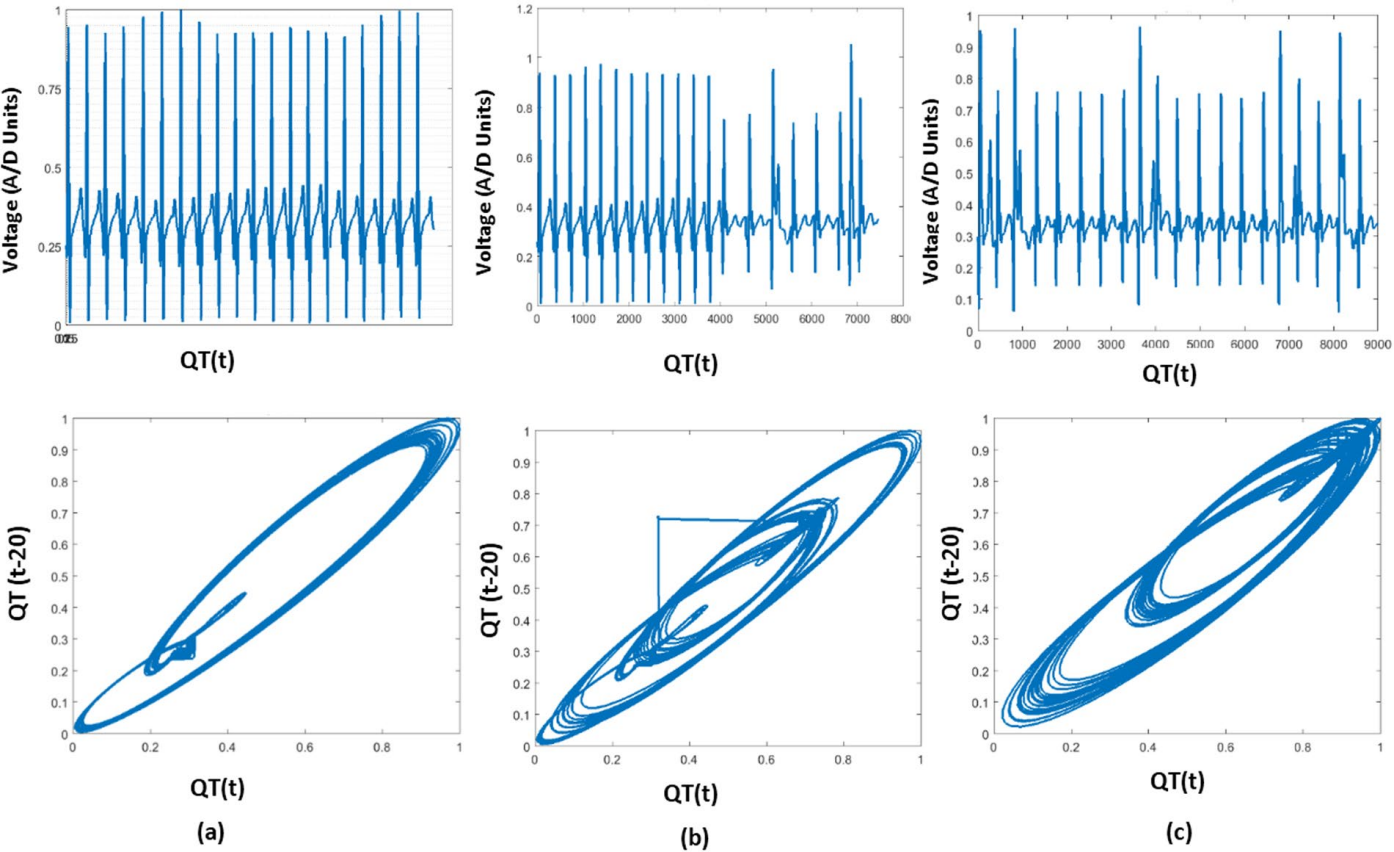
PSR plots of the window occupying QT, (case **a**) Window having only Healthy QT intervals and its PSR plot, (case **b**) Window having a combination of healthy and unhealthy QT intervals and its PSR plot, (case **c**) Window having only unhealthy QT intervals and its PSR plot.

The mathematical equation of the sliding window for the QT interval is shown in the Eq. (9). In the Fig. 14, sliding window (SW_1) moves from the beginning to the end of the array, the window holds only healthy QT intervals in the beginning, the PSR image of the window is generated by plotting the original 20 consecutive QT intervals (SW_QT_ (t)) and the delayed signal (SW_QT_ (t-20)) as shown in Fig. 16(a). After a few slides the window (orange color window in Fig. 14) occupies the mix of healthy and unhealthy QT intervals (Partial unhealthy), moving further, the window (Red color window in Fig. 14) holds only the unhealthy QT interval, the PSR plots of the corresponding sliding windows are shown in the Fig. 16(b,c).

9 $$S{W}_{{\rm{QT}}}=\{QT(i),\,QT(i+\mathrm{1),}\,QT(i+\mathrm{2),}\,\mathrm{....}QT(i+\mathrm{19)}\},\,i\in \{\mathrm{1,}\,\mathrm{2,}\,\mathrm{3...}n-19\}$$

From the visual inspection it is apparent that the PSR plot (Fig. 16) (a)) of healthy QT intervals have fewer trajectories occupied in the image compared to the PSR plots (Fig. 16(b,c)) of unhealthy QT intervals. When the sliding window moves from healthy to partial unhealthy we can observe the change of chaotic motions (Fig. 16) (b)) in the PSR image occupying more boxes than the previous cases. When the sliding window moves to complete unhealthy condition, we can observe even large chaotic changes in the PSR image as shown in Fig. 16, this results in indicating high box-count values compared to the only healthy QT intervals and the mix of healthy and unhealthy QT intervals. Distribution of box count values of all the QT windows corresponding to all the rows of Eq. (8) is shown in Fig. 1(a). It is evident that the box-count values have increased during the sliding window moving from healthy to an unhealthy condition, this motivates us for observing the statistical parameters of the whole QT intervals trace with respect to the values of the box-count of all the images to recognize the abnormal condition. Table 4 and Table 5 describe the pseudo code of the proposed methodology.

**Table 4:** Pseudo code of the proposed methodology to find CV of ECG localized features.

1. Requirement: Find the CV distribution of all the localized features
2. *E*(*t*) = Healthy + Unhealthy ECG signal
3. *E*_*f*_(*t*) = filtering(*E*(*t*))
4. Normalization: *E*_*n*_(*t*) = (*E*_*f*_(*t*) − *E*_*fmin*_(*t*))/(*E*_*fmax*_(*t*) − *E*_*fmin*_(*t*))
5. Extraction of boundaries: *for* i = 1 to *length* (*E*_*n*_(*t*)) {*E*(1), *E*(2), *E*(3), —*E*(*n*),} = *BD*[*E*_*n*_(*t*)] *endfor* where *E*(*n*) is the nth ECG beat.
6. Extracting ECG Features: *for i* = 1 *to n* {*PR*[*i*], *QRS*[*i*], *QT*[*i*]} = *FE*[*E*(*i*)] *endfor*
7. Taking QT interval array for PSR analysis: *QT*(*i*), *i* ∈ {1, 2, 3, ....*n*}
8. Applying Sliding window on QT interval array: *for z* = 1 *to n* − 19 *SW*(*t*) = {*QT*(*z* + 1), *QT*(*z* + 2), *QT*(*z* + 3)....*QT*(*z* + 19)} *PSR*_*Image*(*k*) = *plot*(*SW*(*t*), *SW*(*t* − 20)) *endfor* *SW* = *Sliding Window*.
9. Box-count array: *for x* = 1 *to k* *Box*_*count*_*array*(*x*) = *No*. *ofBlackBoxes*(*PSR*_*image*(*x*)) *endfor*
10. Sliding window on Box-count distribution: *for m* = 1 *to x* − 19 *CV*(*m*) = *CV*(*Box*_*count*_*array*(*m*), *Box*_*count*_*array*(2), *Box*_*count*_*array*(3))...*Box*_*count*_*array*(*m* + 19) *endfor*
11. *for i* = 1 *to m* *Th*_*max*_(*i*) = *max*[*CV*(*i*){1:*a*}] *Th*_*min*1_(*i*) = *min*[*CV*(*i*){*b*:*end*}] *endfor Note*: *point* ‘*a*’ and ‘*b*’ *in CV plot are corresponding to* ‘*Unhealhty QT start*’ *and* ‘*Healthy QT end*’ *windows of* Fig. 14
12. *Th*_*final*_*max*_*QT*_ = *max*(*Th*_*max*_(1), *Th*_*max*_(2), *Th*_*max*_(3)..*Th*_*max*_(*m*)) *Th*_*final*_*min*_*QT*_ = *min*(*Th*_*min*_(1), *Th*_*min*_(2), *Th*_*min*_(3)..*Th*_*min*_(*m*))
13. *Th*_*final*_*min*_*QT*_ and *Th*_*final*_*max*_*QT*_ *are the two thresholds of CV corresponing to QT interval*, the similar procedure is followed for PR interval and QRS complex to find thresholds.

**Table 5:** Continuation of Pseudo code.

1. Classification using the localized features
2. % Conditions to classify PR Interval
3. if *CV*_th_ of PR ≤ 0.068 then
4. print (Healthy PR interval)
5. else if *CV*_th_ of PR > 0.068 or *CV*_th_ *oƒ PR* < 0.1012 then
6. print(PR interval tending to unhealthy)
7. else
8. print(Unhealthy PR interval)
9. % Conditions to classify QRS complex
10. if *CV*_th_ ≤ 0.069 then
11. print(Healthy QRS complex)
12. else if *CV*_th_ > 0.069 *or* *CV*_th_ < 0.083 then
13. print(QRS complex tending to unhealthy)
14. else
15. print(Unhealthy QRS complex)
16. % Conditions to classify QT interval
17. if *CV*_th_ ≤ 0.079 then
18. print(Healthy QT interval)
19. else if *CV*_th_ > 0.079 *or* *CV*_th_ < 0.082 then
20. print(QT interval tending to unhealthy)
21. else
22. print(Unhealthy QT interval)

To understand the regularity of continuous QT intervals and identifying the desynchronization, we have calculated the mean (*μ*), standard deviation (*σ*) and the coefficient variation (*CV* = *σ*/*μ*) on the distribution of box-count values. Considering the 20 box count values as one window and moved stepwise with an overlap of 19 values as shown in the Fig. 1(a), the window value is chosen large such that it allows tracing the intricate details of the box-counting histograms. Here, {*μ*, *σ*} are given by the first and second central moment of the number of black boxes as in Eq. (10).

10 $$\mu ={E}_{n}[{n}_{b}],\sigma =\sqrt{E{[{n}_{b}-\mu ]}^{2}}$$

**Figure 17:**
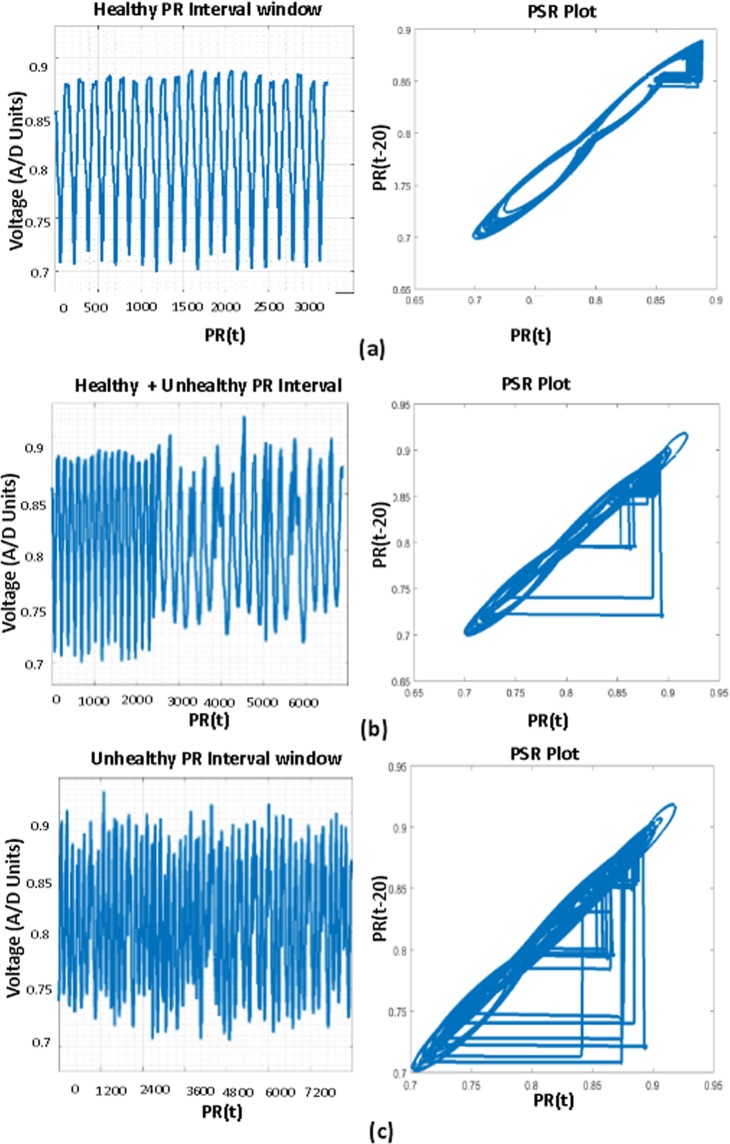
PSR plots of the window covering PR interval. (**a**) Healthy PR intervals and its PSR plot. (**b**) Combination of healthy and unhealthy PR intervals. (**c**) Unhealthy PR intervals and its PSR plot.

**Figure 18:**
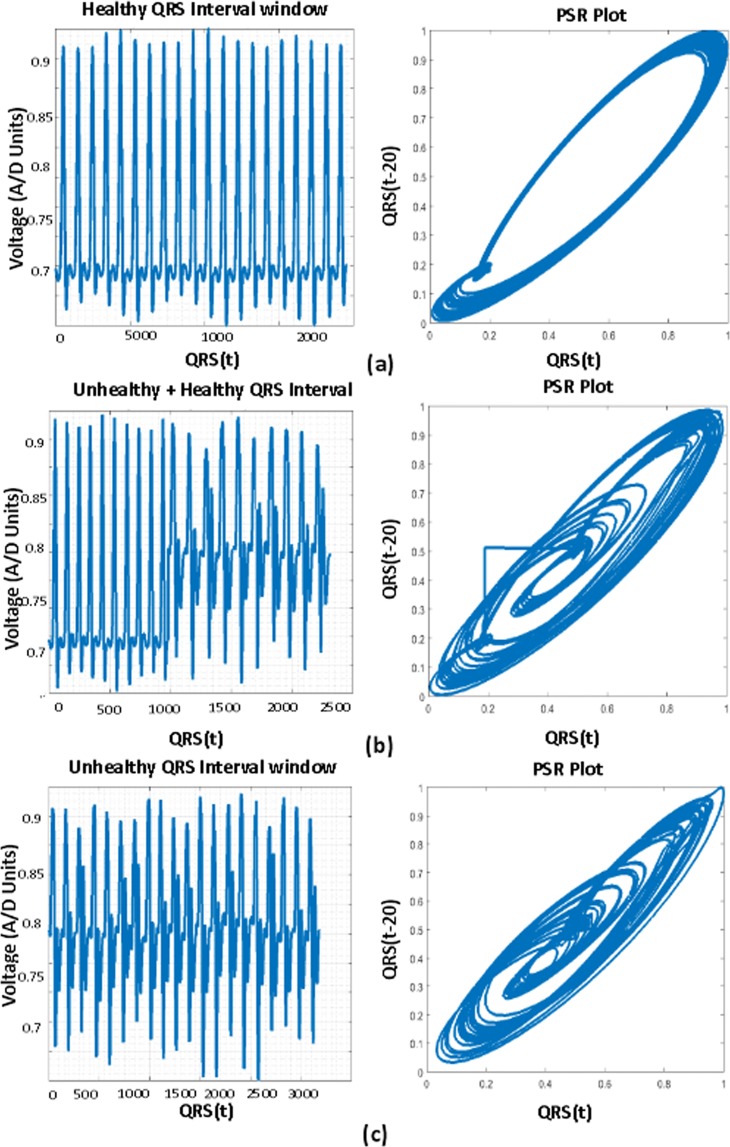
PSR plots of the window covering QRS complexes. (**a**) Healthy QRS complexes and its PSR plot. (**b**) Combination of healthy and unhealthy QRS complexes. (**c**) Unhealthy QRS complexes and its PSR plot.

Figure 2 shows the descriptive statistics mean (*μ*) (Fig. 2(a)), standard deviation (*σ*) (Fig. 2(b)) and coefficient variation (CV = *σ*/*μ*) (Fig. 2(c)) of QT interval, these statistical measures are calculated to know the temporal variations in the box-count distribution graph shown in the Fig. 1, each color in the Fig. 2 corresponds to each patient’s statistics measures. As an example, in the mean distribution graph (Fig. 2(a)), consider the red color line corresponding to a single patient. The first mean value of the red color line is calculated by taking the mean of first 20 image values occupied by the sliding window (green color) in the Fig. 1. When we move the sliding window step wise towards right by one image occupying previous 19 images, the mean of the second window gives the second value of the red color line. Likewise, we calculate the mean, SD and CV for each sliding window and plot the corresponding values shown in Fig. 2. If the number of images in the box-count distribution graph (Fig. 1) are ‘k’, then the number of mean values in the Fig. 2(a) will be ‘k-19’, this is due to the last sliding window as it occupies the last 19 images. Since our proposed work is on the localized features, Fig. 2 gives the mean, SD and CV information corresponding to the localized feature QT interval, whereas Fig. 4 shows the statistics corresponding to the localized features of PR interval and QRS complex respectively. The similar PSR analysis is performed on PR interval and the QRS complex arrays, the corresponding PSR plots are shown in the Figs 17 and 18. The mean, SD and CV (Fig. 4) of PR interval and QRS complexes are calculated to find the abnormalities.

## Data Availability

The datasets analysed during the current study are available in the ‘PhysioNet’, the web address is [https://physionet.org/cgi-bin/atm/ATM].
